# An Approach for a Synthetic CTL Vaccine Design against Zika Flavivirus Using Class I and Class II Epitopes Identified by Computer Modeling

**DOI:** 10.3389/fimmu.2017.00640

**Published:** 2017-06-09

**Authors:** Edecio Cunha-Neto, Daniela S. Rosa, Paul E. Harris, Tim Olson, Alex Morrow, Serban Ciotlos, Charles V. Herst, Reid Martin Rubsamen

**Affiliations:** ^1^Laboratory of Clinical Immunology and Allergy-LIM60, University of São Paulo School of Medicine, São Paulo, Brazil; ^2^Institute for Investigation in Immunology (III) INCT, São Paulo, Brazil; ^3^School of Medicine, Heart Institute (Incor), University of São Paulo, São Paulo, Brazil; ^4^Department of Microbiology, Immunology and Parasitology, Federal University of São Paulo (UNIFESP/EPM), São Paulo, Brazil; ^5^Endocrinology Division, Department of Medicine, School of Medicine, Columbia University, New York, NY, United States; ^6^Flow Pharma, Inc., Redwood City, CA, United States; ^7^Department of Anesthesia, Critical Care and Pain Medicine, Massachusetts General Hospital, Boston, MA, United States

**Keywords:** Zika vaccine, epitope, CTL vaccine, protein folding, dengue, flavivirus, computer model

## Abstract

The threat posed by severe congenital abnormalities related to Zika virus (ZKV) infection during pregnancy has turned development of a ZKV vaccine into an emergency. Recent work suggests that the cytotoxic T lymphocyte (CTL) response to infection is an important defense mechanism in response to ZKV. Here, we develop the rationale and strategy for a new approach to developing cytotoxic T lymphocyte (CTL) vaccines for ZKV flavivirus infection. The proposed approach is based on recent studies using a protein structure computer model for HIV epitope selection designed to select epitopes for CTL attack optimized for viruses that exhibit antigenic drift. Because naturally processed and presented human ZKV T cell epitopes have not yet been described, we identified predicted class I peptide sequences on ZKV matching previously identified DNV (Dengue) class I epitopes and by using a Major Histocompatibility Complex (MHC) binding prediction tool. A subset of those met the criteria for optimal CD8+ attack based on physical chemistry parameters determined by analysis of the ZKV protein structure encoded in open source Protein Data File (PDB) format files. We also identified candidate ZKV epitopes predicted to bind promiscuously to multiple HLA class II molecules that could provide help to the CTL responses. This work suggests that a CTL vaccine for ZKV may be possible even if ZKV exhibits significant antigenic drift. We have previously described a microsphere-based CTL vaccine platform capable of eliciting an immune response for class I epitopes in mice and are currently working toward *in vivo* testing of class I and class II epitope delivery directed against ZKV epitopes using the same microsphere-based vaccine.

## 1 Introduction

As of Fall 2016, the Zika Virus (ZKV) pandemic continues its northward spread in the Americas. The CDC estimates at least 4,100 cases in the United States and up to 29,000 cases in Puerto Rico. Those cases in Puerto Rico include 672 pregnant women (1). Using a data-driven global stochastic epidemic model to project past and future spread of the ZKV in the Americas, it has been estimated that the large population centers of Florida, New York, and New Jersey will be seeing significant numbers of imported cases (acquired by travel) of ZKV infection (2) by the end of Fall 2016. In South America, the new case rate of ZKV infection is tapering off, however, researchers in Brazil warn that official statistics may significantly underestimate the size of the ZKV epidemic based on improved serological tools that have become recently available. In any event, when a significant proportion of the population is infected with a viral infection and become immune, the epidemic can migrate to an area with a larger susceptible individual pool. Given the alarming news that significant brain defects were detected in newborns of 42% women infected with ZKV during pregnancy, including the third trimester (29%) (3), the public health threat of ZKV in pregnant women is even higher than expected before. Taken together, recent estimates put 1.65 million childbearing women in the Americas at risk of ZKV infection. As yet no phase II trials of a ZKV vaccine have been initiated. We review critical aspects of the unique pathogenesis of ZKV infection which will need to be considered when evaluating the efficacy of such vaccines and designing next iterations of possible ZKV vaccines to improve vaccine efficacy. In this article, we will also highlight details of the vaccines currently under consideration for Phase I and Phase II clinical trials, develop the argument that vaccines that evoke antibody responses need careful scrutiny, outline the rationale why our group is focusing on developing a “pure” CTL vaccine, and enumerate many of the challenges that will need to be overcome to develop an effective ZKV CTL vaccine.

### 1.1 Genome and Protein Structure of ZKV

ZKV is a small enveloped plus strand RNA virus belonging to the genus Flavivirus, which includes many human pathogenic viruses, such as Dengue virus (DNV), yellow fever virus (YFV), West Nile Virus (WNV), and hepatitis C virus (HCV). ZKV has a 10.8 kb RNA genome, containing a single open reading frame flanked by a 5′-UTR (106 nt long) and a 3′-UTR (428 nt long). The open reading frame encodes a polyprotein precursor, which is processed into three structural proteins [capsid (C), premembrane (prM), and envelope (E)] and seven non-structural proteins (NS1, NS2A, NS2B, NS3, NS4A, NS4B, and NS5). The viral E protein is the major surface glycoprotein of flavivirus, and the non-structural NS3 and NS5 encode essential enzyme activities for viral reproduction. The E protein is divided into three discernible domains (Domain I, Domain II, and Domain III). Domain I is involved in the envelope structure organization, and Domain II and Domain III are related to the monomers interaction and receptor binding, respectively (4).

### 1.2 Protective Immune Responses to Flaviviruses: Role of T Cells

Significant information is available about the protective role of T cell responses against other flaviviruses of clinical importance. Prevention of infection is achieved primarily by neutralizing antibodies but T cell responses (both CD4+ and CD8+) are of utmost importance for virus clearance. Cytotoxic CD8+ T cells are critical to eliminate virus-infected cells while CD4+ T cells provide help to cytotoxic CD8+ T cells and antibody production (5, 6). DNV-specific CD8+ T cells play a protective role in natural DNV infection both in humans and in animal models (7) and polyfunctional CD8+ responses are associated with protection against disease (8). CD8+ T cell immunity has been shown to be protective against WNV infection (9). Vaccination with a tetravalent DNV vaccine elicits CD8+ T cell responses against highly conserved epitopes (10). Similar, the live-attenuated 17D-based YFV vaccine elicits potent and long-lasting CD8+ T cell responses (11–13). Progress toward understanding the role of CD4+ T cell immunity in flavivirus infection is recent. YFV 17D-204 vaccination and adoptive transfer experiments demonstrate that CD4+ T cells contributed to protection against virulent YFV (14). Similar CD4+ responses have been found to be critical for protection against DNV challenge (15) and for the prevention of encephalitis during WNV infection (16). More recently, the CTL response in a murine ZKV model has shown to be crucial for protection against ZKV infection, both in CD8 depletion experiments in mice and passive transfer of memory CD8+ T cells to naive mice exposed to infection. Furthermore, deletion of the CD8a−/− gene leads to 100% death after infection. This CD8+ T cell response is cytotoxic, polyfunctional, and targeted to several H-2D-restricted epitopes (17).

## 2 Specific Potential Advantages of CTL *Versus* Antibody Vaccine for ZKV

### 2.1 Caveats of Antibody-Inducing ZKV Vaccines

Following the acute phase infection of ZKV (with or without clinical symptoms), the persistence of biomarkers of ZKV infection (e.g., viral RNA in semen) suggest that some cells may be chronically infected. The wide distribution of types and anatomical locations of cells permissive for ZKV infection, sometimes beyond the easy reach of antibodies (e.g., blood–brain barrier), suggest that a cell mediated immune response will be critical for immune surveillance of chronically infected cells. While there can be little doubt that a ZKV vaccine stimulating a neutralizing antibody response will be a key resource in limiting viremia during the acute phase of ZKV infection, there are some concerns regarding the exact nature of the antibody response provoked. The exact pathological mechanism which drives Guillain–Barré syndrome (GBS) remains unknown although there seems to a general consensus that antiglycolipid antibodies play an important role, although not every GBS patient develop this type of antibody. As discussed earlier, there is an increased incidence of GBS associated with ZKV infection (18, 19), but it is not known whether antiganglioside antibodies have a role in this specific comorbidity of ZKV infection. Each of the four different DNV serotypes (DNV 1–4) provoke cross-reactive antibody responses that may contribute to the increased disease severity observed following subsequent infection with a different serotype. The first DNV infection is either subclinical or result in a mild disease, and results in long lasting immunity to the serotype. The next DNV infection, if initiated by a different serotype, can induce severe, potentially lethal disease termed Dengue hemorrhagic fever/Dengue shock syndrome (20, 21). The immunopathogenesis of severe disease is not completely understood. One model, termed antibody-dependent enhancement (ADE), works as follows: anti-DNV antibodies evoked by the primary infection, which were once neutralizing but are not with the current serotype, bind the second serotype viral particles and promote antibody mediated phagocytosis by myeloid antigen-presenting cells which in turn become infected serving as a future reservoir for infectious virions with impaired functional activity (22). Of note are recent reports demonstrating that preexisting anti DNV abs can enhance ZKV infection (23, 24). Conversely, preexisting serum anti-ZKV antibodies were able to enhance DNV infection *in vitro* (25). This is due to the high serological crossreactivity between both flaviviruses which may not be cross-neutralizing. This crossreactivity is so relevant that it has delayed the development of highly specific, non-DNV crossreactive serodiagnostic tests for ZKV infection. An additional concern for flavivirus vaccination-induced pathogenic antibodies in humans came from the recent reports of severe DNV breakthrough infections requiring hospitalization, after vaccination of seronegative volunteers with an antibody-inducing DNV attenuated virus tetravalent vaccine (Dengvaxia^®^), a phenomenon possibly related to ADE (26). This is a special concern since epidemics of both flaviviruses occur simultaneously in the same regions (27). Their research using a mouse model exhibiting much of the same symptoms/pathology of Dengue fever in humans, concluded “a sub-protective humoral response may, under some circumstances, have pathological consequences.” This group has since shifted their focus to inducing CD8+ T cell-mediated immunity to DNV (7, 28–31). Furthermore, the possibility that preexisting non-neutralizing anti-ZKV antibody-dependent enhancement could facilitate infection of fetal–mother interface tissues and contribute to fetal ZKV infection has not been excluded yet. Of note, currently studied ZKV candidate vaccines currently in the pipeline, either in the preclinical or phase I trial (one ongoing trial) phases, aim to elicit antibodies and are all based on whole envelope proteins, or whole inactivated or live attenuated virus (32). Preclinical studies using vaccines encoding whole ZKV preM/E proteins in DNA form, using adenovirus vectors, or whole inactivated ZKV in non-human primate models have been able to elicit neutralizing antibodies and protection after ZKV challenge (33, 34).

Taken together, these findings suggest caution in needed in the development of whole protein ZKV vaccines where evoked antibody responses that are not neutralizing may possibly enhance infection or be pathogenic (i.e., autoimmune) or could facilitate infection of maternal–fetal interface tissue.

### 2.2 Epitope-Based T Cell Vaccines

Given the concerns with antibody-inducing flavivirus vaccines, one possible alternative would be to harness the power of the T cell immune response in protecting against flavivirus infection, as mentioned above. A recent report has shown that CD8+ T cell prevent antigen induced antibody-dependent enhancement of Dengue disease in a murine model and several studies have identified DNV T cell epitopes appropriate for inclusion in a T cell-based vaccine (31, 35–38). Another recent study shows the critical role of CTL response for protection against ZKV infection in a mouse model; this article identifies ZKV H-2D restricted epitopes recognized by CD8+ T cells from infected mice (17). Recent clinical trials have demonstrated the efficacy of T-cell-inducing vaccines against a number of diseases (39), but immunization with whole proteins may favor responses to regions subject to antigenic drift and immune escape. A way to counteract this is to focus the response into specific desirable epitopes. The T cell epitope-based vaccine approach may target the immune response only to desirable and relevant epitopes, instead of the whole protein. Relevant epitopes include those that come from conserved viral protein regions, and/or where mutations could lead to reduced viral fitness, and those that bind to multiple MHC variant molecules—thus potentially recognized by the majority of the target population—while avoiding regions that are poorly immunogenic, variable and subject to antigenic drift, or that could cause a harmful response (40). These targeted immune responses could lead to increased potency, as well as increasing safety (41, 42). There are several ongoing clinical trials of T cell epitope-based Influenza vaccines aiming to be universal vaccines (43). Mapping and selection of potential immunogenic T cell epitopes is a crucial step that may be performed either with the aid of bioinformatics tools and experimental confirmation or by empirical approach using peptide library spanning the antigen full sequence.

### 2.3 Antigenic Drift: Parallels to Chronic HIV Infection and Implications for Vaccine Design

In chronic HIV infection there exists a reservoir of latent, transcriptionally silent viral infection within the resting memory CD4+ T cell compartment and specific myeloid lineage cells (e.g., CD14+/CD16+ monocytes) [reviewed in Ref. (44, 45)]. The resting CD4+ memory cells have long life spans, can remain quiescent, and similar to some of the ZKV tissue targets such placental, neuronal, and gonadal tissues as recently described in mice (46), may reside in immune-privileged sites such as the B cell follicle of lymph nodes, allowing escape from existing immune surveillance mechanisms (47). While the mechanism that triggers active replication in HIV+ CD4+ memory cells is poorly understood, interruption of antiretroviral therapy is associated with the resumption of viral replication. Unfortunately, preexisting HIV-1-specific CD8+ T cell responses have shown to be ineffective [reviewed in Ref. (48)] due to viral evolution of CTL epitopes, resulting in a limited repertoire of effective of cytotoxic T cell-mediated immune responses (49) and progression to AIDS. In HIV infection there are selection pressures exerted by the cellular immune system which result in antigenic drift in new virons (50).

A recent murine model study has demonstrated the potential importance of the CTL response to ZKV infection where H-2D restricted CTL epitopes were identified (17). Studies of HIV specific CTL responses in a subset of HIV+ individuals may also prove informative. HIV controllers (i.e., individuals who are HIV+ yet maintain low viral loads and do not progress to AIDS) have been carefully studied (51). HIV controller status is associated with the ability to develop CTL responses to regions of HIV proteins critical for maintenance of their structure–function (and viral fitness). Pereyra et al. (51) demonstrated that it may be possible to predict CTL class I epitopes favored by HIV controllers and suggested that CTL vaccines designed to evoke cellular immune responses to MHC class I restricted epitopes found within viral protein regions resistant to antigenic drift could lead to improved efficacy of HIV vaccines perhaps mimicking what happens naturally in HIV controllers. Our group has been inspired by these studies and has selected this general approach in the development of a CTL vaccine for ZKV.

Flaviviruses mutate in response to immune system pressure, both by antibodies and T cells. It has been reported that HLA class I-binding residues of a CD8+ T cell epitope encompassing the conserved catalytic site of DNV NS3 protease suffer variation that can abrogate HLA class I binding, suggesting evasion of DNV from a specific CD8+ T cell response by antigenic drift (52). Antigenic drift in ZKV has not been thoroughly studied, but a phylogenetic analysis of contemporary human isolates show a common ancestor and as many as 34 amino acid substitutions relative to the common ancestors with most of the variation contained within the prM protein (53, 54), suggesting that ZKV does not undergo viral evolution as fast as HIV does. However, a recent phylogenetic study on 17 whole ZKV genomes from human isolates in the present epidemic has shown the mutation rate varies between 12 and 25 bases (0.12–0.25% of the polyprotein) per year since the 2013 Polynesia outbreak. The latest sequence shows 64 mutations; and overall, 62 non-synonymous amino acid changes were observed among all sequences analyzed, demonstrating that the ZKV continues to mutate at a rapid rate during the current epidemic (55). The rationale of focusing CTL attack to ZKV protein regions that are “intolerant” to amino acid substitutions thus remains sound.

### 2.4 ZKV HLA Class I Epitope Identification: HLA Binding and Structural Entropy

Human class I epitopes have not yet been formally identified for ZKV. Some authors have published ZKV MHC class II epitope prediction based on MHC binding search engines alone (35, 56, 57). In order to generate a realistic list of MHC-1 binding peptides on ZKV E and M proteins, not only did we use a binding prediction tool, but we also performed matching known DNV class I epitopes to peptides on ZKV. This is warranted due to the antigenic similarity of DNV and ZKV, which display 44–68% sequence identity, as well as the reported crossreactivity to ZKV of DNV envelope-specific antibodies (58). An additional layer of identification was the structural entropy analysis described in the next section.

We generated the predicted ZKV epitope list using the sequence of ZKV Strain H/PF/2013 (GenBank Accession number: KJ776791.2) (59). This strain was isolated from an infected patient during the French Polynesia epidemic in 2013–2014. The E and M protein amino acid sequences were run through the MHC-I Binding Predictions tool available on IEDB (60). This tool combines data from multiple prediction methods, which include artificial neural networks stabilized matrices. Choosing only those alleles that occur in at least 1% of the human population, we generated a list of predicted epitopes for MHC-A and B alleles. Percentile rank is calculated by comparing a given predicted peptide’s IC50 (concentration of the query peptide which inhibits 50% of a reference peptide binding) against those of a random set drawn from (61) where smaller rank indicates higher affinity. The highest ranking MHC-A and -B alleles are presented in Tables 1 and 2.

**Table 1:** Structural entropy (*SE*) calculated by the authors for DNV homologous and/or MHC binding predicted class I epitopes on ZKV E.

Epitope	*SE*	Source	Start	Stop	1° Allele	2° Allele	*%Rank*Δ 1° ↔ 2°
ALGGVLIFL	1.57	Dengue/Predicted	490	498	HLA-A*02	HLA-B*58	6.9
LTMNNKHWLV	1.73	Dengue/Predicted	204	213	HLA-A*02	HLA-B*08	1.45
GLFGKGSLV	1.78	Dengue/Predicted	106	114	HLA-A*02	HLA-B*08	16.7
TMNNKHWLV	1.84	Dengue/Predicted	205	213	HLA-A*02	HLA-B*08	1.55
YYLTMNNKHW	1.86	Dengue/Predicted	202	211	HLA-A*23	HLA-B*53	0.4
QEGAVHTAL	1.89	Dengue/Predicted	261	269	HLA-B*40	HLA-A*32	23.8
AVHTALAGA	1.96	Dengue/Predicted	264	272	HLA-A*30	HLA-B*07	1.6
YSLCTAAFTF	1.98	Dengue/Predicted	305	314	HLA-A*23	HLA-B*53	0.3
KEWFHDIPL	2.08	Dengue/Predicted	215	223	HLA-B*40	HLA-A*02	8.3
SQILIGTLLM	2.09	Dengue/Predicted	464	473	HLA-B*15	HLA-A*26	2.25
SYSLCTAAF	2.10	Dengue/Predicted	304	312	HLA-A*23	HLA-B*15	2.4
TPHWNNKEAL	2.13	Dengue/Predicted	233	242	HLA-B*07	HLA-A*23	46.25
ILIGTLLMW	2.13	Dengue/Predicted	466	474	HLA-B*57	HLA-A*33	42.65
DTAWDFGSV	2.13	Dengue/Predicted	426	434	HLA-A*68	HLA-B*51	12.8
LALGGVLIF	2.14	Dengue/Predicted	489	497	HLA-B*53	HLA-A*23	1.5
HKEWFHDIPL	2.15	Dengue/Predicted	214	223	HLA-B*40	HLA-A*32	0.45
MAVLGDTAW	2.16	Dengue/Predicted	421	429	HLA-B*53	HLA-A*32	5.2
RMAVLGDTAW	2.17	Dengue/Predicted	420	429	HLA-B*58	HLA-A*24	3.9
ILIGTLLMWL	2.17	Dengue/Predicted	466	475	HLA-A*02	HLA-B*15	5.5
VSYSLCTAAF	2.18	Dengue/Predicted	303	312	HLA-A*24	HLA-B*15	1.25
RLKGVSYSL	2.20	Dengue/Predicted	299	307	HLA-A*32	HLA-B*08	0.3
FKSLFGGMSW	2.28	Dengue/Predicted	453	462	HLA-B*58	HLA-A*23	3.5
KSLFGGMSW	2.28	Dengue/Predicted	454	462	HLA-B*57	HLA-A*32	0.15
KMMLELDPPF	2.33	Predicted	373	382	HLA-A*02	HLA-B*44	0.4
EFKDAHAKR	2.61	Dengue/Predicted	244	252	HLA-A*33	HLA-B*08	57.8

*Start-Stop positions are relative to the ZKV E protein. %*Rank*Δ reflects the absolute value of the difference between the best fit, primary (1°) and secondary (2°) allele percentile rank. Larger numbers for %*Rank*Δ indicate a larger preference for the 1° allele*.

**Table 2:** Structural entropy (*SE*) calculated by the authors for DNV homologous and/or MHC binding predicted class I epitopes on ZKV M.

Epitope	*SE*	Source	Start	Stop	1° Allele	2° Allele	*%Rank*Δ 1° ↔ 2°
VMILLIAPA	1.83	Predicted	65	74	HLA-A*30	HLA-B*15	0.45
LVMILLIAPA	1.87	Dengue/Predicted	64	73	HLA-A*02	HLA-B*08	2.75
YLVMILLIA	1.89	Dengue/Predicted	63	71	HLA-A*02	HLA-B*35	1.75
VMILLIAPAY	1.93	Predicted	65	74	HLA-A*30	HLA-B*15	0.45
IYLVMILLI	2.02	Dengue/Predicted	62	70	HLA-A*23	HLA-B*51	0.2
ALAAAAIAWL	2.24	Predicted	43	52	HLA-A*02	HLA-B*15	4.2
SQKVIYLVM	2.26	Dengue/Predicted	58	66	HLA-B*15	HLA-A*30	3.8
LLGSSTSQKV	2.25	Dengue/Predicted	52	61	HLA-A*02	HLA-B*51	17.35
TSQKVIYLV	2.33	Dengue/Predicted	56	65	HLA-A*68	HLA-B*57	0.55
LIRVENWIFR	2.37	Dengue/Predicted	29	38	HLA-A*31	HLA-B*57	31.25
VTLPSHSTR	2.58	Predicted	2	11	HLA-A*11	HLA-B*57	6.55
LPSHSTRKL	2.65	Predicted	3	12	HLA-B*07	HLA-A*02	4.65
SQTWLESREY	2.79	Predicted	16	25	HLA-B*15	HLA-A*30	1.45
RSQTWLESR	2.81	Predicted	15	23	HLA-A*31	HLA-B*57	6.25
KLQTRSQTW	2.84	Predicted	11	19	HLA-A*32	HLA-B*57	0.2

*Start-Stop positions are relative to the ZKV M protein. %*Rank*Δ reflects the absolute value of the difference between the best fit, primary (1°), and secondary (2°) allele percentile rank. Larger numbers for %*Rank*Δ indicate a larger preference for the 1° allele*.

In order to maximize matching known DNV class I epitopes against the ZKV sequences, we were indiscriminate with respect to the DNV strain sequences. We used epitope sequence data from all of DNV strains 1–4, as downloaded from IEDB. Alignments between predicted ZKV epitopes and DNV were calculated using MAFFT (62) and webPRANK (63).

A recent study by Stettler (58) indicated ZKV/DNV cross-recognition observed for antibodies may not also be present for T-cell epitopes. Because more work is needed on this topic, and in order to analyze a larger set of potential ZKV epitopes, the class I epitopes listed in Tables 1 and 2 are initially predicted, and only afterward aligned to DNV. Allowing for sequence divergence between DNV and ZKV, as well as keeping in mind the antigenic divergence between strains of ZKV, we did not require strict conservation between the predicted ZKV epitopes and the DNV epitopes they were compared to. As such, non-homologous but predicted epitopes were included in these tables. There are no table entries for epitopes matched to DNV but not predicted. Reported HLA specificities refer specifically to ZKV epitope predictions.

#### 2.4.1 Computing Structural Entropy to Select Class I Epitopes for a CTL Vaccine

X-ray crystallography can be used to generate a PDB file containing a complete mathematical representation of the three-dimensional properties of a protein (64). Software is available which can take a PDB file as input and predict changes in the protein’s three-dimensional structure after specified amino acid substitutions. One example of such a program is FoldX, which compute whole-protein free energy changes resulting from these specified amino acid changes (65).

Pereyra-Heckerman described an index they call structural entropy (*SE*) which codifies the extent to which a free energy change will occur after CTL escape at that epitope (51). A low *SE* indicates that at least one amino acid position in an epitope, a relatively high change in the protein’s free energy is expected to occur after mutations to one or more amino acids in that epitope. They analyzed class I epitope targets preferred by HIV controllers and reported that these individuals have a statistically significant preference to attack class I epitopes associated with a low *SE*.

Identifying *SE* may be a key criterion for picking class I epitope CTL attack points especially for vaccines targeting pathogens that exhibit viral escape due to antigenic drift. Designing vaccines that focus cellular immunity toward these structurally critical regions of proteins may prove advantageous. Pereyra-Heckerman’s observation that HIV controllers preferentially target epitopes with a low *SE* suggests that it may be possible to design vaccines based entirely on an *in silico* analysis of the protein structure.

*SE* is calculated using a four-step process. First, a 20-element free energy change vector is created for each amino acid position within each class I epitope codifying the free energy change computed after each of all possible 20 amino acid substitutions at that amino acid position. Note that one of those substitutions will be the amino acid for itself resulting in a free energy change of “zero” for one of the entries in the 20-element free energy change vector. Second, each 20-element free energy change vector associated with each amino acid in the epitope is converted into a 20 element Boltzmann probability distribution. A particularly high Boltzmann distribution entry indicates that there is a relatively high probability of that particular amino acid occurring at that position. The first and second steps are implemented in equation (1) taken from Pereyra-Heckerman. Third, each Boltzmann distribution is converted into a single Shannon entropy value shown in Pereyra-Heckerman equation (2). A Low Shannon entropy indicates a Boltzmann distribution with at least one entry significantly higher than the others, indicating a relatively high preference for the wild type. Fourth, the *SE* is calculated by taking the mean of the Shannon entropies for the individual amino acids in the epitope.

(1) $$p_{\mathrm{ij}}=E|f_{\mathrm{ij}}|\;=\frac{e^{-|\mathrm{dd}G_{\mathrm{ij}}|}}{\sum_{k=1}^{20}\;e^{-|\mathrm{dd}G_{\mathrm{ik}}|}}$$

(2) $$E|H_{i}|\;=-\sum_{j=1}^{20}\;p_{\mathrm{ij}}\;\ln\;(p_{\mathrm{ij}})$$

In systems with many possible energy states, the Boltzmann distribution is typically used to compute the relative probability of each energy state occurring. The Boltzmann distribution is used here, in the context of amino acid substitutions, to estimate the probability of each substitution occurring by considering how much each substitution changes the protein’s energy relative to the wild type. We assume that the wild type is the most likely state, and compute the Boltzmann distribution from the free energy changes relative to the wild type, generating a distribution of probabilities for each substitution. Amino acids that do not cause large energy changes will have a high probability, large-valued entry in the Boltzmann distribution, and mutations which cause large energy changes will have low Boltzmann values. We can think of the values in the Boltzmann Distribution as measuring the “naturalness” of each mutation at the given site.

The ZKV structural entropy data was generated using the PDB file (64) uploaded to RCSB by Sirohi et al. in March of 2016 (66). Protein structure data was available for ZKV E and M proteins only. The original DNA sequence used to generate this protein structure was based on the ZKV Strain H/PF/2013 (GenBank Accession number: KJ776791.2) (59). The ZKV E protein has been identified as the main source of H-2D-restricted MHC class I epitopes recognized by CD8+ T cells from ZKV-infected mice (17).

**Figure 1:**
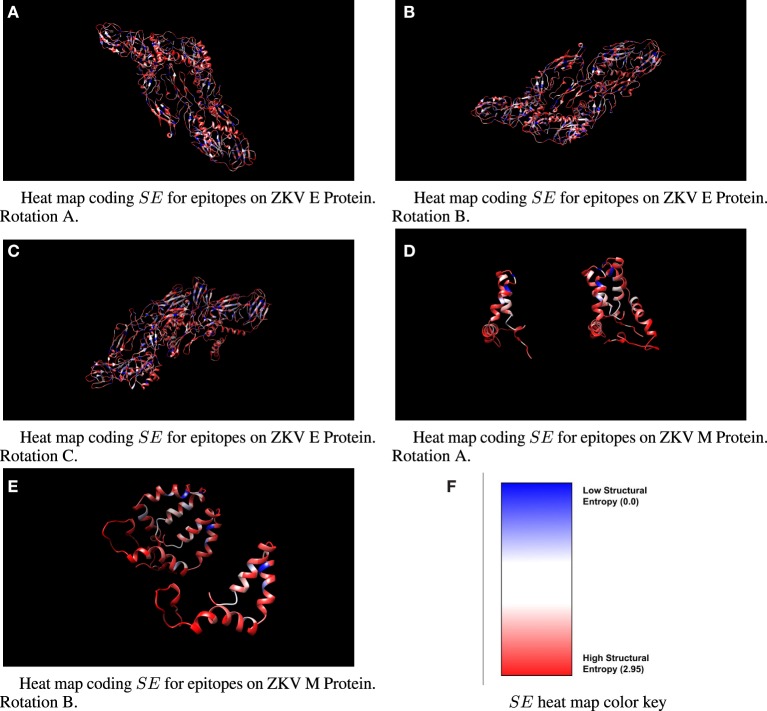
Three-dimensional ribbon view of ZKV E and M with moving-window-calculated *SE* values shown in heat map format. Note that the color coded *SE* regions do not represent *SE* values for specific epitope sequences.

*SE* data for class I epitopes identified on ZKV E are shown in Table 1 and for ZKV M in Table 2. Qualitative heat maps showing *SE* values computed using moving windows across all amino acids in ZKV E and ZKV M are shown in Figure 1. These heat maps are not based on the specific epitope sequences identified in the tables. They are qualitative and are intended to show the distribution of *SE* values throughout the proteins. Note that low *SE* regions, shown in blue, are in the minority. By raking the epitopes in order of *SE* in Tables 1 and **2**, we list the epitopes predicted to be the best CTL targets based on Pereyra-Heckerman first.

### 2.5 Mapping of Potential Epitopes in ZKV Capable of Binding to Multiple HLA Class II Molecules

The rational selection of CD4+ T cell epitopes in vaccine formulation is crucial for successful application of vaccination strategies that focus on induction of CD8+ T cell immunity, given the role of CD4+ T cell response in long-term maintenance of CD8+ T cell-dependent protective immunity. Recently, CD4+ T cells with cytotoxic features have been identified in PBMC from patients with chronic viral infections (67–70). Bioinformatics tools for identification of HLA class II epitopes have been reviewed by Ref. (71). The TEPITOPE HLA-DR binding prediction algorithm (72) and the derived ProPred algorithm (73) use the concept that each HLA-DR pocket in the antigen-binding groove can be characterized by “pocket profiles,” a quantitative representation of the interaction of all natural amino-acid residues with a given pocket, creating a matrix incorporated in the TEPITOPE and ProPred softwares. For each HLA-DR specificity, the algorithms generated a binding score corresponding to the algebraic sum of the strength of interaction between each residue and pocket, which correlated with binding affinity. Peptide scores along a scanned protein sequence are normalized for each HLA-DR as the proportion of the best binder peptides (74). Since the software predicts binding to a significant number of HLA-DR specificities (25 in the case of TEPITOPE, 51 for PROPRED), it is also capable of predicting promiscuous peptide ligands each capable of binding to multiple HLA class II variant molecules (58). The TEPITOPE prediction algorithm has been successfully applied to the identification of dozens of promiscuous T cell epitopes frequently recognized in 59 antigenic proteins from several human pathogens including viruses, bacteria, protozoa, fungi, and helminths (HIV, SIV, CMV. *M. tuberculosis, P. vivax, P. brasiliensis, S. mansoni*), and *in silico* prediction correlated with promiscuity in HLA-binding assays and frequency of T cell recognition by exposed individuals (75). This has led to several epitope-based vaccines which were shown to be immunogenic using conventional or and HLA class II-transgenic mice (71, 76) and protective (77) in mice. The incorporation of a promiscuous CD4+ T cell epitope in a recombinant protein-based *P. vivax* vaccine led to significant increase in its immunogenicity (41). A recent study from our group in non-human primates showed that a HIV CD4+ T cell epitope-based DNA vaccine was highly immunogenic and induced significant responses to most encoded epitopes in all animals tested (unpublished observations). Vaccines encoding promiscuous peptides able to bind to multiple HLA-DR molecules may thus allow wide population coverage. Here, we used the TEPITOPE and ProPred algorithms to identify potential “promiscuous” CD4+ T cell epitopes—predicted to bind to multiple HLA-DR molecules—derived from conserved regions of ZKV majority/consensus E and M protein sequences from circulating strains in the recent epidemic in Brazil and Polynesia.

### 2.6 Selection of ZKV Sequences and Promiscuous HLA Class II Epitope Prediction

**Figure 2:**
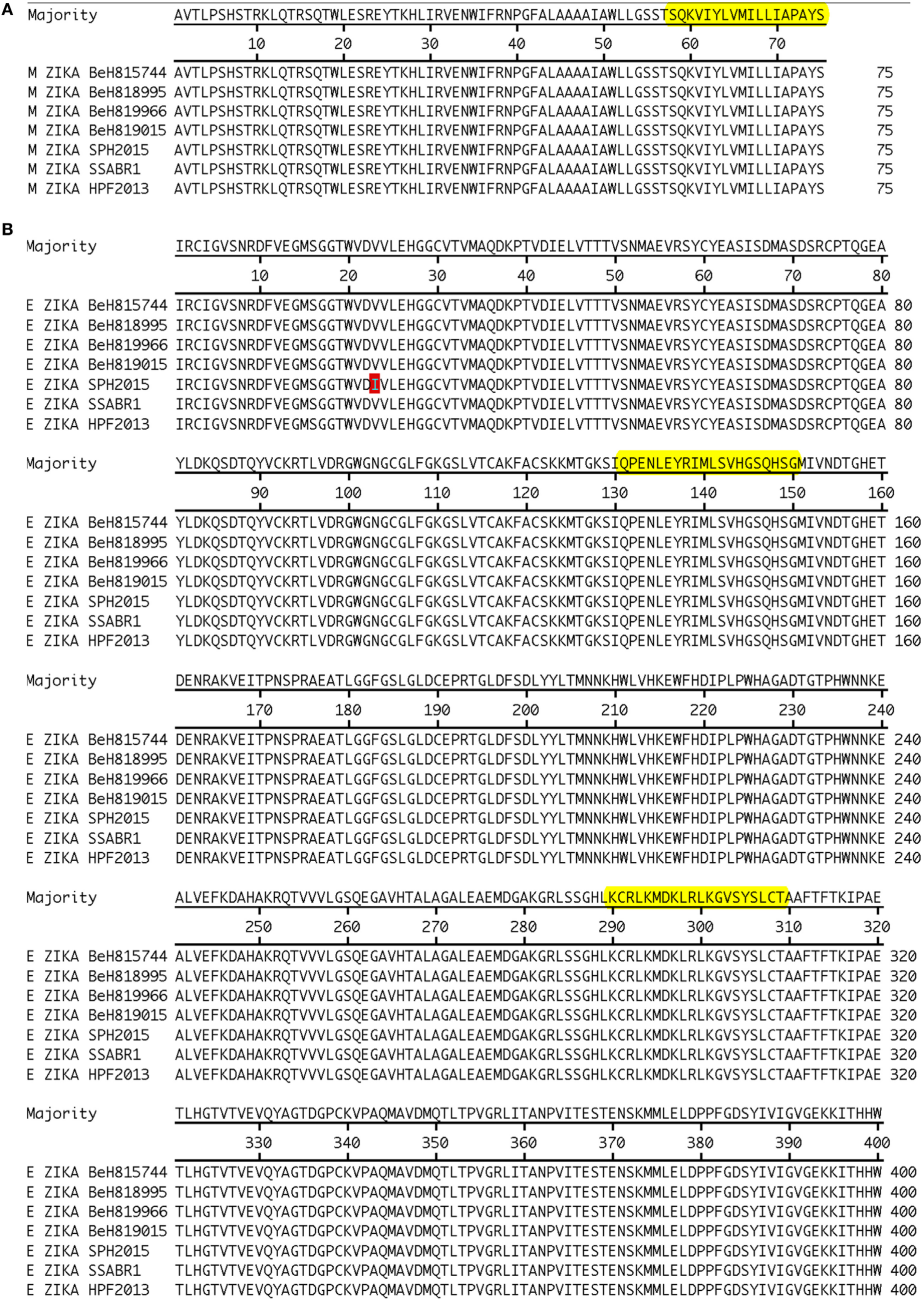
Alignment of ZKV M **(A)** and E **(B)** proteins with epitope identification.

The amino acid sequences derived from the ZKV strains BeH818995 (Genbank accession number KU365777.1), BeH819015 (Genbank accession number KU365778.1), BeH815744 (Genbank accession number KU365780.1), BeH819966 (Genbank accession number KU365779.1), SPH2015 (Genbank accession number KU321639.1), and SSABR1 (Genbank accession number KU707826.1), isolated in Brazil; and the H/PF/2013 strain (Genbank accession number KJ776791.2) isolated in French Polynesia were assembled and aligned with Clustal W (MegAlign, DNASTAR, Madison, WI, USA, Figure 2). We scanned the generated consensus sequence with the TEPITOPE and ProPred algorithms. We selected ZKV M and E peptides (Table 3) whose sequences were predicted to bind to at least 2/3 out of the 25 or 51 HLA-DR molecules in the TEPITOPE or ProPred matrixes, respectively, corresponding to an inner nonamer core selected as the HLA binding motif with flanking amino acids added when possible at either or both N- and C-terminal ends, to increase the efficiency of *in vitro* peptide presentation to CD4+ T cells.

**Table 3:** Model-predicted class II epitopes on ZKV E and ZKV M proteins.

Epitope ID	Epitope sequence	Interacting HLA-DR alleles	% predicted/51
M (58–77)	SQKVIYLVMILLIAPAYSIR	DRB1*0101, DRB1*0102, DRB1*0301, DRB1*0305, DRB1*0306, DRB1*0307, DRB1*0308, DRB1*0309, DRB1*0311, DRB1*0401, DRB1*0402, DRB1*0404, DRB1*0405, DRB1*0408, DRB1*0410, DRB1*0421, DRB1*0423, DRB1*0426, DRB1*0701, DRB1*0703, DRB1*0801, DRB1*0802, DRB1*0804, DRB1*0806, DRB1*0813, DRB1*0817, DRB1*1101, DRB1*1102, DRB1*1104, DRB1*1106, DRB1*1107, DRB1*1114, DRB1*1120, DRB1*1121, DRB1*1128, DRB1*1301, DRB1*1302, DRB1*1304, DRB1*1305, DRB1*1307 DRB1*1311, DRB1*1321, DRB1*1322, DRB1*1323, DRB1*1327, DRB1*1328, DRB1*1501, DRB1*1502, DRB1*1506, DRB5*0101, DRB5*0105	100
E (130–149)	QPENLEYRIMLSVHGSQHSG	DRB1*0101, DRB1*0102, DRB1*0301, DRB1*0305, DRB1*0309, DRB1*0401, DRB1*0402, DRB1*0404, DRB1*0405, DRB1*0408, DRB1*0410, DRB1*0421, DRB1*0423, DRB1*0426, DRB1*0701, DRB1*0703, DRB1*0801, DRB1*0802, DRB1*0804, DRB1*0806, DRB1*0813, DRB1*0817, DRB1*1101, DRB1*1102, DRB1*1104, DRB1*1106, DRB1*1107, DRB1*1114, DRB1*1120, DRB1*1121, DRB1*1128, DRB1*1301, DRB1*1302, DRB1*1304, DRB1*1305, DRB1*1307, DRB1*1311, DRB1*1321, DRB1*1322, DRB1*1323, DRB1*1327, DRB1*1328, DRB1*1501, DRB1*1502, DRB1*1506, DRB5*0101, DRB5*0105	92
E (289–308)	KCRLKMDKLRLKGVSYSLCT	DRB1*0301, DRB1*0305, DRB1*0306, DRB1*0307, DRB1*0308, DRB1*0309, DRB1*0311, DRB1*0401, DRB1*0402, DRB1*0404, DRB1*0405, DRB1*0408, DRB1*0410, DRB1*0421, DRB1*0423, DRB1*0426, DRB1*0801, DRB1*0802, DRB1*0804, DRB1*0806, DRB1*0813, DRB1*0817, DRB1*1101, DRB1*1102, DRB1*1104, DRB1*1106, DRB1*1107, DRB1*1114, DRB1*1120, DRB1*1121, DRB1*1128, DRB1*1301, DRB1*1302, DRB1*1304, DRB1*1305, DRB1*1307, DRB1*1311, DRB1*1321, DRB1*1322, DRB1*1323, DRB1*1327, DRB1*1328, DRB5*0101, DRB5*0105	86

### 2.7 Potential Synthetic CTL Vaccine Platforms for Class I and Class II Epitope Delivery

H-2D-restricted class I epitopes, when injected intradermally without adjuvants, produce a weak immune response in C57BL/6 mice. Methods have been described for eliciting immune responses to class I. For example, the target epitopes are linked together as a “string of beads” (78). In another example, the DNA corresponding to the desired string of epitopes is inserted in a modified vaccinia Ankara (MVA) vector. Immune responses have been elicited in mice using this technique (79). A DNA string has also been administered with electroporation (80). Immune responses in Macaques have been elicited in this manner (81). In order to add and subtract epitopes from the formulation used in these types of vaccines, new linker elements must be identified and proper presentation of the desired epitopes after “string-of-beads” processing by antigen-presenting cells confirmed (82).

The use of a biodegradable, PLGA microsphere-based vaccine delivery platform allows one or more unmodified peptides to easily be incorporated into the vaccine formulation (83). The limitations of PLGA microsphere-based vaccines have been described in the literature. For example, double-emulsion sphere fabricating technologies may degrade the tertiary structure of the delivered antigen due to exposure to solvents or high temperatures used during spray drying processes (84). In a previous report, we manufactured our microspheres avoiding double emulsion sphere manufacturing technology using a precision spray drying process that operates at room temperature (85).

In contrast to previous studies which incorporated only a single peptide epitope in spheres (86), we showed that it was possible to elicit an immune response from each of two epitopes delivered simultaneously, when the two epitopes were loaded into the same spheres or different spheres.

This is an important consideration, especially because the HLA restricted nature of the class I epitopes being delivered will require the development of a “master vaccine” containing enough different peptide epitopes to cover a target population.

The fact that the majority of the epitopes listed in the first four rows of Tables 1 and 2 have the best predicted HLA match as HLA*02 suggests that a vaccine directed against these class I epitopes could readily tested in Brazil where the frequency of HLA A*02 frequency varies from 21.7 to 47.5% between states (87).

## 3 Concluding Remarks

The search for rapid development of safe and effective vaccines against ZKV is a global public health emergency. Testing multiple vaccine platforms in parallel may speed up and increase the likelihood of finding a good vaccine. We have proposed a rationale for ZKV epitope selection and design of T cell epitope-based vaccine against ZKV virus. Selection of candidate ZKV structure-constrained HLA class I epitopes able to bind an array of HLA class I supertypic molecules, and promiscuous class II T cell epitopes capable of binding to multiple HLA class II molecules could provide wide HLA and population coverage for such a vaccine which could be delivered using the synthetic, adjuvanted microsphere vaccine as outlined above or other techniques for epitope immunization that we discussed.

## Ethics Statement

This study was carried out in accordance with the policies and procedures established by the WIRB Institutional Review Board with written informed consent from all subjects. All subjects gave written informed consent in accordance with the Declaration of Helsinki. The protocol was approved by the WIRB.

## Author Contributions

RR, EC-N, DR, and PH wrote the manuscript. All authors reviewed the manuscript. All authors directly participated in the research described with EC-N and DR performing modeling predictions of class II epitopes and AM, TO, SC, and RR performing class I epitope identification and *SE* calculations for class I epitopes.

## Conflict of Interest Statement

RR is CEO of Flow Pharma, Inc. RR, AM, TO, SC, and CH have received compensation in the form of cash and/or stock from Flow Pharma, Inc. All other authors declare that the research was conducted in the absence of any commercial or financial relationships that could be construed as a potential conflict of interest.
